# Enhancing fever of unknown origin diagnosis: machine learning approaches to predict metagenomic next-generation sequencing positivity

**DOI:** 10.3389/fcimb.2025.1550933

**Published:** 2025-04-15

**Authors:** Zhi Gao, Yongfang Jiang, Mengxuan Chen, Weihang Wang, Qiyao Liu, Jing Ma

**Affiliations:** ^1^ Department of Infectious Diseases, The Second Xiangya Hospital, Central South University, Changsha, China; ^2^ FuRong Laboratory, Changsha, Hunan, China; ^3^ Clinical Research Center For Viral Hepatitis In Hunan Province, Changsha, Hunan, China

**Keywords:** metagenomic next-generation sequencing (mNGS), fever of unknown origin (FUO), machine learning algorithms, light gradient-boosting machine (LightGBM), predictive modeling

## Abstract

**Objective:**

Metagenomic next-generation sequencing (mNGS) can potentially detect various pathogenic microorganisms without bias to improve the diagnostic rate of fever of unknown origin (FUO), but there are no effective methods to predict mNGS-positive results. This study aimed to develop an interpretable machine learning algorithm for the effective prediction of mNGS results in patients with FUO.

**Methods:**

A clinical dataset from a large medical institution was used to develop and compare the performance of several predictive models, namely eXtreme Gradient Boosting (XGBoost), Light Gradient-Boosting Machine (LightGBM), and Random Forest, and the Shapley additive explanation (SHAP) method was employed to interpret and analyze the results.

**Results:**

The mNGS-positive rate among 284 patients with FUO reached 64.1%. Overall, the LightGBM-based model exhibited the best comprehensive predictive performance, with areas under the curve of 0.84 and 0.93 for the training and validation sets, respectively. Using the SHAP method, the five most important factors for predicting mNGS-positive results were albumin, procalcitonin, blood culture, disease type, and sample type.

**Conclusion:**

The validated LightGBM-based predictive model could have practical clinical value in enhancing the application of mNGS in the etiological diagnosis of FUO, representing a powerful tool to optimize the timing of mNGS.

## Introduction

In the past 50 years, sequencing technology has undergone significant development, leading to significant improvements in speed, accuracy and cost-effectiveness and enabling researchers to explore the complexity of the genome in unprecedented detail (Heather and Chain, 2016). First-generation sequencing methods, predominated by Sanger sequencing, played an important role in the early stage of genomics, considering their advantages in sequencing small DNA fragments. The completion of the Human Genome Project in 2003 marked a key step in the field, leading to the development of next-generation sequencing (NGS) technologies. Corresponding applications greatly increased the throughput and reduced the costs of DNA sequencing, making it possible to sequence the entire genome in a timely and economical manner (Mardis, 2011). The landscape of sequencing technologies has constantly evolved since the emergence of NGS. The latest advancement is long-read sequencing technology, which enables the sequencing of longer continuous DNA fragments. It offers a major solution for deciphering complex genomic regions and understanding structural variations that were previously difficult to analyze (Goodwin et al., 2016). In addition to providing broader access to genomic data by the public, the continuous advancement of sequencing technologies also provides new paths for research and clinical applications, especially concerning personalized medicine (Casey et al., 2013). The integration of sequencing technology into clinical practice has upgraded medical diagnosis and treatment modes, highlighting the profound impact of these advances on healthcare.

Metagenomic NGS (mNGS) has become the most commonly used high-throughput sequencing technology for detecting pathogenic microorganisms and avoiding limitations related to traditional culture-based methods. This innovative strategy can comprehensively identify various pathogens directly in clinical specimens without bias in the absence of prior knowledge of specific pathogens (Simner et al., 2018). Advancements in mNGS technologies have significantly promoted its application in clinical microbiology to achieve rapid and accurate diagnoses of infectious diseases. mNGS can also identify common and rare pathogens, leading to enhanced practicality in clinical settings, as timely and accurate diagnosis is crucial for effective patient management (d'Humières et al., 2021). mNGS displays excellent sensitivity in detecting bacteria, fungi, parasites, viruses, and some specific pathogens. Using this method, researchers can comprehensively analyze microbial communities and identify pathogens that might have been missed by traditional culture-based strategies. mNGS can support the analysis of complicated samples without prior knowledge of the pathogenic type, highlighting its potential use in clinical diagnosis (Han et al., 2019). Moreover,compared with traditional blood culture, the sensitivity of mNGS is less affected by antibiotic use, maintaining high detection rates even upon significant reductions in the density of pathogenic bacteria (Caliendo and Hodinka, 2017; Miao et al., 2018). To further validate this claim, we conducted a subgroup analysis to compare mNGS positivity rates between patients with prior antibiotic use and those without antibiotic exposure. Among the 284 enrolled patients, 174 (61.3%) received antibiotics prior to mNGS testing, while 110 (38.7%) did not. The mNGS positivity rates in these two groups were 62.1% (108/174) and 67.3% (74/110), respectively. Aχ²test revealed no statistically significant difference between these groups (χ²= 0.812, P = 0.368).

These findings support the hypothesis that prior antibiotic exposure does not significantly affect mNGS sensitivity, consistent with previous studies. The ability of mNGS to detect microbial nucleic acids even in patients with reduced bacterial loads highlights its advantage over traditional culture-based methods, which are more susceptible to antibiotic interference.

Consequently, mNGS technologies have been extensively applied in the diagnosis of infectious diseases, displaying advantages such as high positivity rates, minimal interference from antibiotics, and wide pathogen coverage (Caliendo and Hodinka, 2017). In clinical settings, traditional microbiological testing, pathological examination, and sterile specimen culture remain the gold standards for infection diagnosis. Notably, mNGS has been accepted as a powerful complement that further expands the scope and depth of pathogen identification and provides new paths for the diagnosis of complicated infections.

More than 1,000 pathogenic microorganisms can induce disease development in humans. However, for certain infectious diseases (e.g., intracranial infections, bloodstream infections), traditional microbiological testing fails to provide a clear pathogenic diagnosis in >50% of cases. This poses a great challenge to the application of traditional methods in the etiological identification of complicated infections, thereby increasing the requirement for more sensitive and comprehensive detection techniques to improve pathogenic identification rates (Glaser et al., 2006). Biological diagnostic methods are affected by bacterial density and antibiotic use, and the test is time-consuming (≥48 h), which could delay the diagnosis. Moreover, many pathogens are difficult or impossible to culture, prompting clinicians to use broad-spectrum antibiotics without identifying the pathogen. This both imposes an additional financial burden on patients and promotes the development of multidrug resistance in pathogenic microorganisms, further hindering the application of anti-infectious therapy (Liu and Ma, 2024). Technology that can identify potential pathogens by detecting microbial nucleic acids (i.e., NGS, mNGS) in multiple samples of culture-negative patients has proven effective for microbial identification. However, the sensitivity and specificity of mNGS varied in different studies. In one study, the sensitivity and specificity of mNGS were 50.7% and 85.7%, respectively (Miao et al., 2018). However, mNGS outperforms traditional culture-based methods in identifying specific pathogens such as *Mycobacterium tuberculosis*, viruses, anaerobic bacteria, and fungi (Miao et al., 2018). Consequently, the diagnostic potential of mNGS in complex microbial cases, especially culture-negative cases, has emerged (Hu et al., 2019).

Nevertheless, mNGS can generate negative results in the clinical detection of fever of unknown origin (FUO), which might be attributable to non-infectious diseases or sample types. In cases of local infection, mNGS using tissue displayed better positivity rates and sensitivity than blood culture (Ramachandran and Wilson, 2020). Conversely, the sensitivity of mNGS was lower than that of blood culture for specific pathogens such as *Brucella* species, which are gram-negative bacteria that can survive and multiply within their host by evading the immune response (von Bargen et al., 2012). Therefore, clinicians should conduct comprehensive analyses based on patients’ clinical manifestations, laboratory test results and medical history. This could optimize the allocation of medical resources and reduce the financial burden on patients.

Machine learning (ML) is a subset of artificial intelligence in which machines autonomously acquire information by extracting patterns from large databases (Ermak et al., 2024), and it has been increasingly used within the medical community. Among the ML algorithms, eXtreme Gradient Boosting (XGBoost) is a decision tree-based ensemble boosting algorithm framework with high training efficiency and accuracy, and it is especially suitable for handling imbalanced datasets. Light Gradient-Boosting Machine (LightGBM) is a highly efficient gradient-boosting algorithm with faster training speed and lower memory consumption that can be used to process large-scale data. Random Forest (RandomForest) is an ensemble learning method that improves the stability and accuracy of a model by constructing multiple decision trees and integrating corresponding prediction results.

Feature selection and model evaluation are crucial when applying these ML algorithms. Feature selection is beneficial for identifying target variables with the greatest impact on prediction results, and model evaluation ensures the reliable performance of the selected model in practical applications (Chen et al., 2020). ML methods are increasingly used in genomics, especially in fields such as variation detection and functional annotation (Zou et al., 2019). The proposed algorithms can improve researchers’ understanding of the potential biological significance of genomic data, in addition to improving the predictive accuracy (Schreiber and Singh, 2021).

Compared to the general advancements in sequencing technologies, the clinical value of metagenomic next-generation sequencing (mNGS) is particularly prominent in the context of fever of unknown origin (FUO) diagnosis. FUO remains a diagnostic dilemma characterized by prolonged fever without an identified cause after standard clinical evaluation. Traditional diagnostic workflows, relying heavily on blood cultures, serology, and imaging, often yield inconclusive results, with approximately half of FUO cases lacking a confirmed etiology. In this scenario, mNGS serves as a transformative tool by enabling the comprehensive and unbiased detection of pathogens from clinical specimens, bypassing the limitations of culture-based methods. Its ability to identify rare, fastidious, and atypical organisms makes mNGS an essential complement to conventional diagnostics in FUO cases. Furthermore, mNGS shows superior diagnostic performance in patients with prior antibiotic exposure, where traditional blood cultures are frequently rendered negative. Thus, focusing on the application of mNGS in FUO is of critical importance to optimize pathogen detection, improve patient management, and reduce the burden of empirical treatment.

Based on the selection of appropriate features to construct a predictive model using ML methods, this study aimed to predict mNGS-positive results using specimens from patients with FUO using XGBoost, LightGBM, and RandomForest.

## Methods

This retrospective cohort study enrolled patients with FUO admitted to a large comprehensive hospital (Grade III Level A) in China. The protocol was approved by the Ethics Committee of Second Xiangya Hospital of Central South University, and all participants signed informed consent forms. All procedures performed in this study were in strict accordance with the ethical standards of the Declaration of Helsinki.

To further assess the generalizability of our model, we implemented an expanded validation strategy using 5-fold cross-validation. This method ensures that each sample is used for both training and validation, reducing the risk of biased performance estimates due to a single train-test split.

In the cross-validation process, the dataset was randomly partitioned into five equal subsets, with the model being trained on four subsets and evaluated on the remaining one in an iterative manner. The mean AUC across all folds was 0.559, with a standard deviation of 0.075, indicating consistent performance across different data splits.

The inclusion of cross-validation strengthens the reliability of our findings by demonstrating that the model maintains stable performance across different dataset partitions. This additional validation step reinforces the model’s applicability to broader clinical scenarios, reducing concerns about overfitting to a specific validation set.

To further assess the generalizability of our model, we implemented an expanded validation strategy using 5-fold cross-validation. This method ensures that each sample is used for both training and validation, reducing the risk of biased performance estimates due to a single train-test split.

In the cross-validation process, the dataset was randomly partitioned into five equal subsets, with the model being trained on four subsets and evaluated on the remaining one in an iterative manner. The mean AUC across all folds was 0.559, with a standard deviation of 0.075, indicating consistent performance across different data splits.

The inclusion of cross-validation strengthens the reliability of our findings by demonstrating that the model maintains stable performance across different dataset partitions. This additional validation step reinforces the model’s applicability to broader clinical scenarios, reducing concerns about overfitting to a specific validation set.

To ensure the validity of our data imputation strategy, we conducted Little’s MCAR (Missing Completely at Random) test to statistically assess the randomness of missing data. The test yielded a p-value of 0.217, indicating that the missing data do not significantly deviate from a completely random distribution. This result supports our assumption that the data are Missing at Random (MAR), justifying the use of multiple imputation techniques.

However, if the assumption of MAR were incorrect, the missing data mechanism could introduce systematic bias, potentially affecting the robustness of our predictive model. For example:

If data were Not Missing at Random (NMAR), systematic differences between observed and missing values could lead to biased estimations of key predictive features, affecting model performance.

Under such conditions, standard imputation methods might fail to accurately recover the missing values, necessitating alternative approaches such as pattern-mixture models or sensitivity analyses to assess the potential impact of missing data on prediction accuracy.

To mitigate these risks, we ensured that imputed values were consistent with observed distributions and verified that model performance remained stable before and after imputation. This additional step reinforces the robustness and reliability of our findings.

### Participants

The cohort included 284 patients diagnosed with FUO (14–88 years old, in stable condition) who were admitted to the Infectious Disease Department of a large Grade III teaching hospital in China from May 2020 to August 2023. The inclusion criteria were as follows: a medical diagnosis of FUO, receipt of mNGS, available blood culture results, complete data, and length of hospital stay >1 day. The exclusion criteria were as follows: fever caused by malignancies of the hematological system, fever induced by *M. tuberculosis* infection, and >30% missing data.

### Data and clinical information collection

This study collected clinical characteristics and auxiliary laboratory examination data, including sex, age, mNGS results, sample type, pathogen type, disease type, antibiotic use, infection site, imaging features (such as liver and spleen enlargement), blood culture, inspection indicators, course of disease, total medical expenses, length of hospital stay, and specimen submission interval.

To mitigate the potential impact of class imbalance on model training, we employed several strategies:

Weighted Loss Function: In tree-based models (LightGBM, XGBoost), we assigned class weights inversely proportional to class frequencies, ensuring that the minority class (negative mNGS cases) was not underrepresented in learning.

Synthetic Data Generation (SMOTE): To verify model stability, we applied Synthetic Minority Over-sampling Technique (SMOTE) in an auxiliary experiment, artificially generating samples for the minority class, and observed that model performance remained stable.

Stratified Sampling: We used stratified train-test splitting to preserve the original class distribution in both training and validation sets, preventing biases during model evaluation.

These techniques ensured that the model was not overly biased toward the majority class while maintaining high generalizability.

### Processing of missing data

As a retrospective study, it was impossible to collect all clinical items because of individual differences in clinical practice. To reduce the impact of missing data, multiple imputation was performed, and all missing data were missing at random. Meanwhile, to ensure data integrity, data imputation in this study was completed using the fully conditional specification method based on “IterativeImputer” in the scikit-learn learning repository (version 1.0.0) of Python (version 3.7.1, Python Software Foundation, Wilmington, DE, USA).

To address missing data, this study applied a multiple imputation strategy based on the fully conditional specification (FCS) method using the “IterativeImputer” module from scikit-learn. The FCS method iteratively models each variable with missing values conditional on other variables, providing a robust approach for datasets where the missingness mechanism is assumed to be Missing at Random (MAR). We conducted Little’s MCAR test to verify this assumption, yielding a p-value of 0.217, supporting the MAR hypothesis and the suitability of multiple imputation.

To evaluate the impact of imputation on model performance, we compared model outputs before and after imputation using LightGBM, XGBoost, and RandomForest models. Without imputation, models exhibited reduced AUCs in both training and validation sets, with performance metrics dropping by an average of 6–8%, particularly in the LightGBM model (pre-imputation AUC: 0.865 vs. post-imputation AUC: 0.932). This suggests that imputation contributed significantly to enhancing model generalizability and discriminative power by addressing information loss due to incomplete data.

Additionally, we performed sensitivity analyses by comparing SHAP value distributions before and after imputation. Results showed that feature importance rankings remained largely consistent, indicating that the imputation strategy did not introduce systematic bias or distort model interpretability. This reinforces the reliability of the imputed dataset and confirms that the chosen strategy effectively minimized potential adverse effects associated with missing data, thus contributing to model robustness.

### Feature selection

The least absolute shrinkage and selection operator (LASSO) method was used to select and screen features to avoid over-fitting of the models. Five key features were ultimately selected through LASSO regression analysis. On this basis, Shapley additive explanation (SHAP) analysis was employed to evaluate the significance of these five variables to quantify the impact of each feature on the model’s prediction results.

LASSO was chosen for feature selection due to its ability to perform both variable selection and regularization simultaneously through L1 penalty, effectively reducing overfitting. Unlike mutual information or forward selection, LASSO automatically removes irrelevant features, enhances model interpretability, and efficiently handles multicollinearity. Additionally, it is computationally efficient and integrates seamlessly into the model training process, making it more suitable for high-dimensional clinical datasets. The selected features were then used in LightGBM and XGBoost models to optimize mNGS positivity prediction.

### Sensitivity analysis

To assess the robustness of selected features, we conducted a sensitivity analysis by systematically excluding one key feature at a time and evaluating the impact on model performance. Using Gradient Boosting Classifier, we retrained the model after removing each feature and compared the AUC with the full model (AUC = 0.511). The results are as follows:

ALB removed → AUC = 0.538

PCT removed → AUC = 0.473

Blood Culture removed → AUC = 0.486

Disease Type removed → AUC = 0.453

Sample Type removed → AUC = 0.460

The AUC dropped most significantly when Disease Type and Sample Type were removed, suggesting their critical role in predicting mNGS positivity. Conversely, ALB showed a minor increase in AUC, potentially due to interactions with other features. These findings confirm that the selected features contribute meaningfully to model predictions, reinforcing the model’s robustness.

### Interpretable ML tools

The interpretation of the predictive model was achieved using SHAP analysis, a unified technique that can accurately quantify the contribution and role of each feature in the final prediction results (Lundberg and Lee, 2017). The application of the model in this study was displayed through a nomogram, in which the SHAP values of each sample were visualized to improve the interpretability and transparency of the model.

### Statistical analysis

All statistical analyses and calculations were conducted using R software (The R Foundation for Statistical Computing, Vienna, Austria) and Python (3.8.0). The specific R packages used in this study included glmnet to implement the LASSO method, rms to draw the nomogram and perform calibration curve analysis, and rmda to implement decision curve analysis (DCA) and plot the clinical impact curve. Categorical variables were expressed as absolute numbers and percentages, and inter-group differences were compared using the χ^2^ test or Fisher’s exact test when the expected frequency was lower than 10. Continuous variables were presented as the median and interquartile range, and Wilcoxon’s rank-sum test was used for inter-group comparisons. The Wilcoxon and Kruskal–Wallis H tests were used to compare the statistical differences in categorical demographic variables among the enrolled patients. Furthermore, univariate and multivariate linear regression analyses were conducted to identify potential factors associated with delayed diagnosis. Significance was denoted by P < 0.05.

XGBoost, LightGBM, and RandomForest were used to develop predictive models, considering their confirmed effectiveness in modeling and predicting results (Wiemken and Kelley, 2020). The predictive performance of each model was visualized using the receiver operating characteristic (ROC) curve and the corresponding area under the curve (AUC) to quantify its discriminating power. Moreover, DCA was applied to evaluate the predictive value of each model at different threshold probabilities to measure the actual utility of the model during decision-making. Simultaneously, calibration curves for the three models were plotted to evaluate their calibration, that is, the consistency between the predictive probability of the model and the actual probability of occurrence. To further demonstrate the effectiveness of each predictive model in actual clinical or application environments, clinical impact curves were drawn to evaluate their impact on clinical treatment decision-making.

To assess the variability of feature contributions and enhance the interpretability of the SHAP analysis, we incorporated bootstrap-based confidence intervals into the SHAP importance plot. Specifically, we performed 1,000 bootstrap resampling iterations on the dataset. In each iteration, SHAP values were recalculated, and the mean SHAP value along with its 95% confidence interval was computed for each feature. This approach enabled us to quantify the uncertainty associated with each feature’s importance and ensure a more robust interpretation of the model outputs.

To assess model calibration, we computed the calibration curve and performed a linear regression fit on the predicted versus observed probabilities. The slope and intercept of the calibration curve were 0.064 and 0.426, respectively, indicating that while the model exhibits reasonable calibration, slight deviations exist at certain probability ranges.

Furthermore, we evaluated model calibration using the Brier score, which measures the accuracy of probabilistic predictions. Our model achieved a Brier score of 0.289, suggesting a good balance between reliability and sharpness in probability estimates.

For clinical impact analysis, we selected a decision threshold of 0.5, based on common clinical practices and statistical considerations (e.g., maximizing Youden’s index). This threshold allows for an optimal trade-off between sensitivity and specificity, ensuring practical applicability in mNGS positivity prediction.

To further evaluate model performance, we included precision, recall, F1-score, and Matthews correlation coefficient (MCC) in addition to the AUC. On the validation set, the model achieved a precision of 0.839, recall of 0.800, F1-score of 0.819, and MCC of 0.522, indicating a strong balance between sensitivity and specificity. The MCC value further supports the model’s robustness by accounting for class imbalance, demonstrating its reliable predictive performance.

To optimize the performance of each machine learning model, we conducted hyperparameter tuning using a grid search strategy combined with 5-fold cross-validation. For each model (LightGBM, XGBoost, and Random Forest), independent sets of hyperparameters were systematically tested to identify the optimal configuration.

For LightGBM, the grid search included parameters such as:

learning_rate: [0.01, 0.05, 0.1]

num_leaves: [31, 63, 127]

max_depth: [5, 10, 15, -1]

min_data_in_leaf: [20, 50, 100]

feature_fraction: [0.6, 0.8, 1.0]

bagging_fraction: [0.6, 0.8, 1.0]

lambda_l1 and lambda_l2: [0, 0.1, 1.0]

For XGBoost, the following hyperparameters were tuned:

learning_rate: [0.01, 0.05, 0.1]

max_depth: [3, 6, 9]

subsample: [0.6, 0.8, 1.0]

colsample_bytree: [0.6, 0.8, 1.0]

gamma: [0, 0.1, 0.5]

reg_alpha and reg_lambda: [0, 0.1, 1.0]

For Random Forest, the grid search included:

n_estimators: [100, 200, 500]

max_depth: [None, 10, 20, 30]

min_samples_split: [2, 5, 10]

min_samples_leaf: [1, 2, 4]

max_features: [‘sqrt’, ‘log2’, None]

Each model’s hyperparameters were tuned separately using scikit-learn’s GridSearchCV function (for Random Forest and XGBoost) or LightGBM’s built-in parameter search function, based on maximizing the area under the ROC curve (AUC) as the primary evaluation metric. The best-performing hyperparameter set for each model was subsequently used to train the final version of the respective model on the training dataset.This systematic tuning process ensured that each model was evaluated under optimized conditions, enhancing fairness in comparative performance assessment.

To ensure model robustness and mitigate overfitting, we implemented a 5-fold cross-validation (CV) strategy during model development. The dataset was randomly partitioned into five equal subsets, with each fold serving as a validation set once while the remaining four folds were used for training. This process was repeated iteratively for each model (LightGBM, XGBoost, Random Forest), ensuring that every patient sample contributed to both model training and validation.

The use of 5-fold CV provided two main advantages:

Overfitting prevention: By repeatedly training and validating across multiple data splits, we minimized the risk of the models learning noise or patterns specific to a single data partition. The average performance across folds offered a more reliable estimate of each model’s generalization capacity.

Robustness assessment: The consistency of the models’ AUCs and other metrics (precision, recall, F1-score) across different folds was used to evaluate model stability. For instance, the LightGBM model exhibited a mean AUC of 0.84 (SD = 0.03) across the folds, indicating minimal variance and good robustness.

Additionally, we ensured that the class distribution (mNGS-positive vs. mNGS-negative) was preserved within each fold via stratified sampling, which further reduced the risk of performance inflation caused by class imbalance.

This cross-validation process supplemented our independent hold-out validation set and served as an additional safeguard against overfitting, reinforcing the reliability of the model before downstream clinical deployment.

## Results

**Figure 1:**
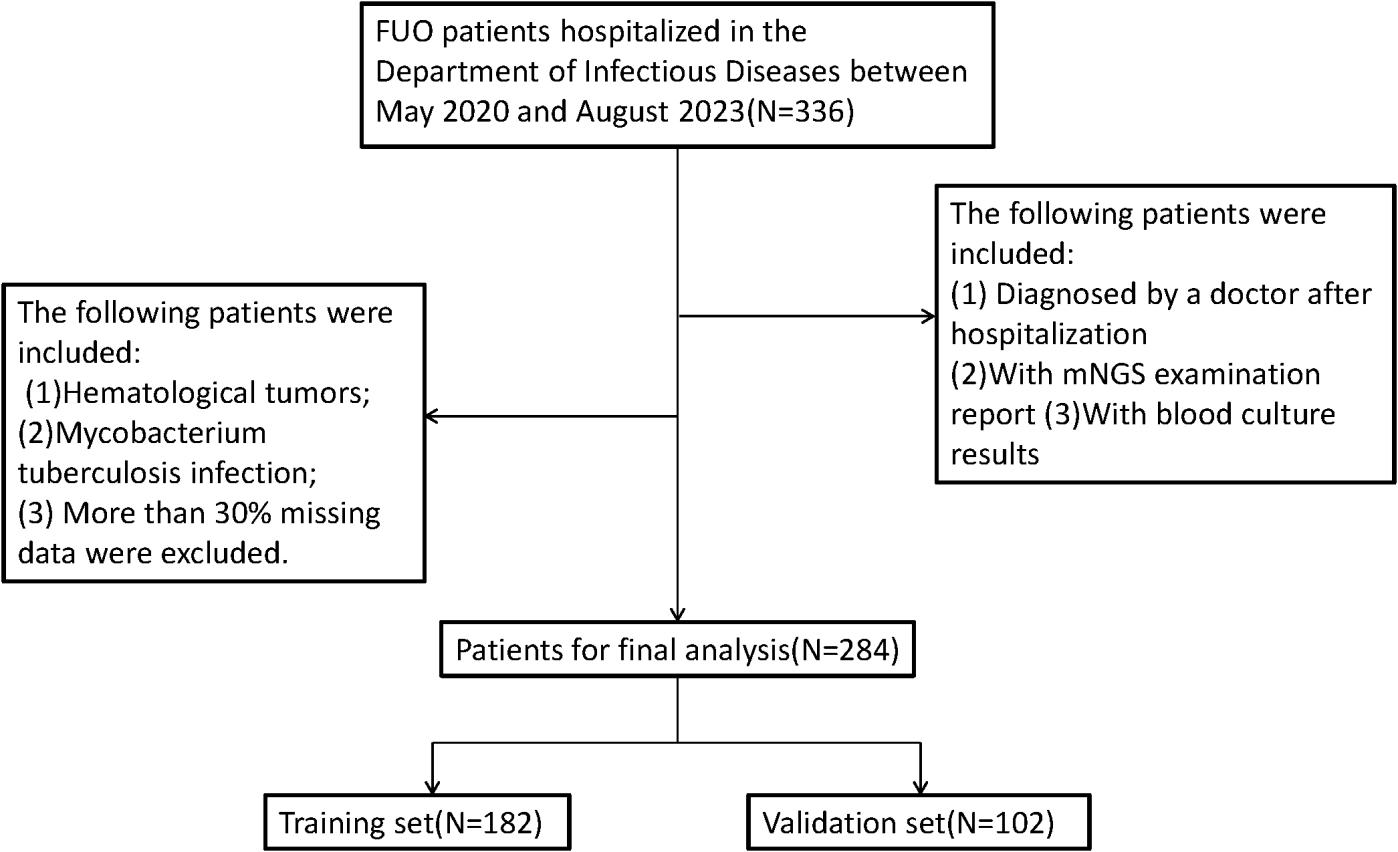
Flow diagram of eligible participants.

The specific research process is presented in **Figure 1**. This figure illustrates the patient selection process for the study. A total of 336 FUO patients hospitalized between May 2020 and August 2023 were initially considered. Patients who met the inclusion criteria (hospitalized diagnosis, availability of mNGS and blood culture results) were included, while those with hematological malignancies, tuberculosis infections, or >30% missing data were excluded. The final dataset consisted of 284 patients, randomly split into a training set (N=182) and a validation set (N=102) for model development and evaluation.Based on the mNGS results, the participants were assigned to the mNGS-positive (n = 182) and or mNGS-negative group (n = 102). Meanwhile, 39 features were incorporated for preliminary analysis, including demographic data, sample type, disease type, antibiotic use, comorbidity, and laboratory tests (**Tables 1**, **2**). Significant differences in sample type, disease type, and bacterial culture results were observed between the groups (**Table 1**), in addition to differences in procalcitonin (PCT), albumin (ALB), and blood Na+ levels (**Table 2**). Univariate logistic regression analysis of the mNGS results revealed significant differences in pathogen type, imaging features (liver and spleen enlargement), alanine aminotransferase (ALT) and aspartate aminotransferase (AST) levels between the groups (all P < 0.05, **Table 3**). Multivariate logistic regression analysis indicated that ALT and AST levels, blood culture, and disease type were significantly correlated with the mNGS results (all P < 0.05, **Table 3**).

**Table 1:** Descriptive statistics of the inpatients’ demographic characteristics.

Characteristics	ALL	NGS		P
		Negative	Positive	
Age (years)^*^	50.85±18.07	48.35±20.71	52.25±16.30	>0.05
Sex, n (%) Female Male	105 (36.97) 179 (63.03)	41 (40.20) 61 (59.80)	64 (35.16) 118 (64.84	>0.05
Specimen type, n (%) Blood Local organization Both	56 (19.72) 220 (77.46) 8 (2.82)	11 (10.78) 90 (88.24) 1 (0.98)	45 (25.73) 130 (71.43) 7 (3.85)	0.002
Pathogenic types, n (%) None Bacteria Virus Fungus Parasite multiple infections	102 (35.92) 40 (14.08) 77 (27.11) 3 (1.06) 2 (0.70) 60 (21.13)	102 (100)	0(0) 40 (31.25) 77 (60.16) 3 (2.34) 2 (1.56) 6 (4.69)	–
Disease type, n (%) Uninfectious Infectious	66 (23.24) 218 (76.76)	36 (35.29) 66 (64.71)	30 (16.48) 152 (83.52)	<0.001
Antibiotic use, n (%) No Yes	64 (22.54) 220 (77.46)	24 (23.53) 78 (76.47)	40 (21.98) 142 (78.02)	>0.05
Infection site, n (%) Local whole	235 (82.75) 49 (17.25)	84 (82.35) 18 (17.65)	151 (82.97) 31 (17.03)	>0.05
Hepatosplenomegaly, n (%) No Yes	199 (70.07) 85 (29.93)	74 (72.55) 28 (27.45)	125 (68.68) 57 (31.32)	>0.05
Blood culture, n (%) Negative Positive None	125 (44.01) 38 (13.38) 121 (42.61)	56 (54.90) 8 (7.84) 38 (37.25)	69 (37.91) 30 (16.48) 83 (45.60)	0.011
Course of disease (days)^*^	41.89±75.93	34.47±55.41	46.05±85.16	>0.05
Hospital costs(10,000 yuan)^*^	37648±49420	30975±50263	41388±48682	>0.05
Hospital day(days)^*^	16.63±13.36	14.48±9.34	17.83±15.05	0.042

*Chi-square test.

**Table 2:** Descriptive statistics of inpatient laboratory indicators.

Test items	ALL patients	Negative patients	Positive patients	P-Value
CRP	81.75±63.15	72.73±56.95	86.80±65.99	>0.05
WBC	9.99±7.09	10.45±9.04	9.74±5.72	>0.05
RBC	3.60±0.83	3.71±0.88	3.53±0.79	>0.05
PLT	244.66±148.72	260.73±140.03	235.65±153.01	>0.05
Neu	7.95±6.33	8.39±7.84	7.71±5.31	>0.05
Lym	1.45±2.43	1.34±1.56	1.52±2.80	>0.05
MCV	90.03±7.75	89.86±8.28	90.13±7.45	>0.05
MCH	29.38±3.27	29.36±3.20	29.38±3.31	>0.05
ESR	63.13±37.15	62.70±36.58	63.37±37.56	>0.05
PCT	3.46±13.09	2.08±11.01	4.23±14.10	<0.001
PT	15.22±6.28	14.52±2.09	15.61±7.67	>0.05
D-DIMER	4.81±14.05	3.96±4.75	5.29±17.19	>0.05
ALT	60.55±88.24	45.53±40.69	68.96±105.13	>0.05
AST	54.88±72.09	43.78±34.45	61.10±85.76	>0.05
ALB	32.57±6.19	34.97±6.90	31.23±5.31	<0.001
GLO	30.58±6.27	30.42±5.14	30.67±6.84	>0.05
BUN	5.91±4.64	5.25±3.64	6.28±5.09	>0.05
CR	89.90±91.93	77.50±37.01	96.85±110.96	>0.05
UA	249.04±120.10	247.40±127.56	249.97±116.06	>0.05
LDH	331.83±256.17	328.15±268.19	333.89±249.92	>0.05
CK	74.21±197.12	81.16±237.07	70.31±171.32	>0.05
CKMB	15.11±14.74	15.03±20.08	15.16±10.72	>0.05
NA+	136.16±4.09	136.82±3.54	135.79±4.33	0.012
TG	1.64±2.42	1.35±0.56	1.80±2.99	>0.05
FBS	5.65±2.28	5.48±1.54	5.75±2.61	>0.05
Ferritin	2789.92±5955.16	2357.87±4833.35	3032.06±6499.93	>0.05
lactic acid	2.64±1.02	2.60±0.96	2.67±1.05	>0.05

Neu, neutrophil; Lym, lymphocytecount; TG, triglycerides; FBS, blood sugar.

**Table 3:** Logistic regression risk factors for NGS results.

Type	Variable	OR	95% CI Lower	95% CI Upper	P-value
Uni	gender	1.055	0.936	1.188	>0.05
	age	1.003	0.999	1.006	>0.05
	Sample type	0.909	0.794	1.041	>0.05
	Pathogen species	1.277	1.117	1.459	<0.01
	Disease type	0.965	0.837	1.112	>0.05
	Antibiotic use	0.997	0.855	1.163	>0.05
	Infection site	0.956	0.842	1.085	>0.05
	Hepatosplenomegaly	1.084	1.019	1.153	<0.05
	Blood culture	1.000	0.999	1.001	>0.05
	CRP	0.994	0.986	1.001	>0.05
	WBC	0.963	0.899	1.031	>0.05
	RBC	1.000	0.999	1.000	>0.05
	PLT	0.993	0.985	1.002	>0.05
	Neu	1.008	0.986	1.03	>0.05
	Lym	1.002	0.994	1.009	>0.05
	MCV	1.000	0.984	1.017	>0.05
	MCH	0.999	0.998	1.001	>0.05
	ESR	1.002	0.997	1.006	>0.05
	PCT	1.005	0.997	1.013	>0.05
	PT	1.002	0.998	1.005	>0.05
	D-DIMER	1.001	1.000	1.001	>0.05
	ALT	1.001	1.000	1.002	<0.05
	AST	0.984	0.974	0.993	<0.01
	ALB	1.001	0.991	1.01	>0.05
	GLO	1.012	0.999	1.025	>0.05
	BUN	1.001	1.000	1.001	>0.05
	CR	1.000	0.999	1.000	>0.05
	UA	1.000	1.000	1.000	>0.05
	LDH	1.000	1.000	1.000	>0.05
	CK	1.000	0.996	1.003	>0.05
	CKMB	0.994	0.98	1.008	>0.05
	NA+	1.016	0.996	1.037	>0.05
	TG	0.996	0.962	1.031	>0.05
	FBS	1.000	1.000	1.000	>0.05
	Ferritin	0.99	0.936	1.048	>0.05
	Lactic acid	1.055	0.936	1.188	>0.05
Mul	ALB	0.985	0.976	0.994	<0.01
	AST	1.001	1.000	1.002	<0.05
	Blood culture	1.097	1.035	1.164	<0.05
	Disease type	1.29	1.132	1.468	<0.01

**Figure 2:**
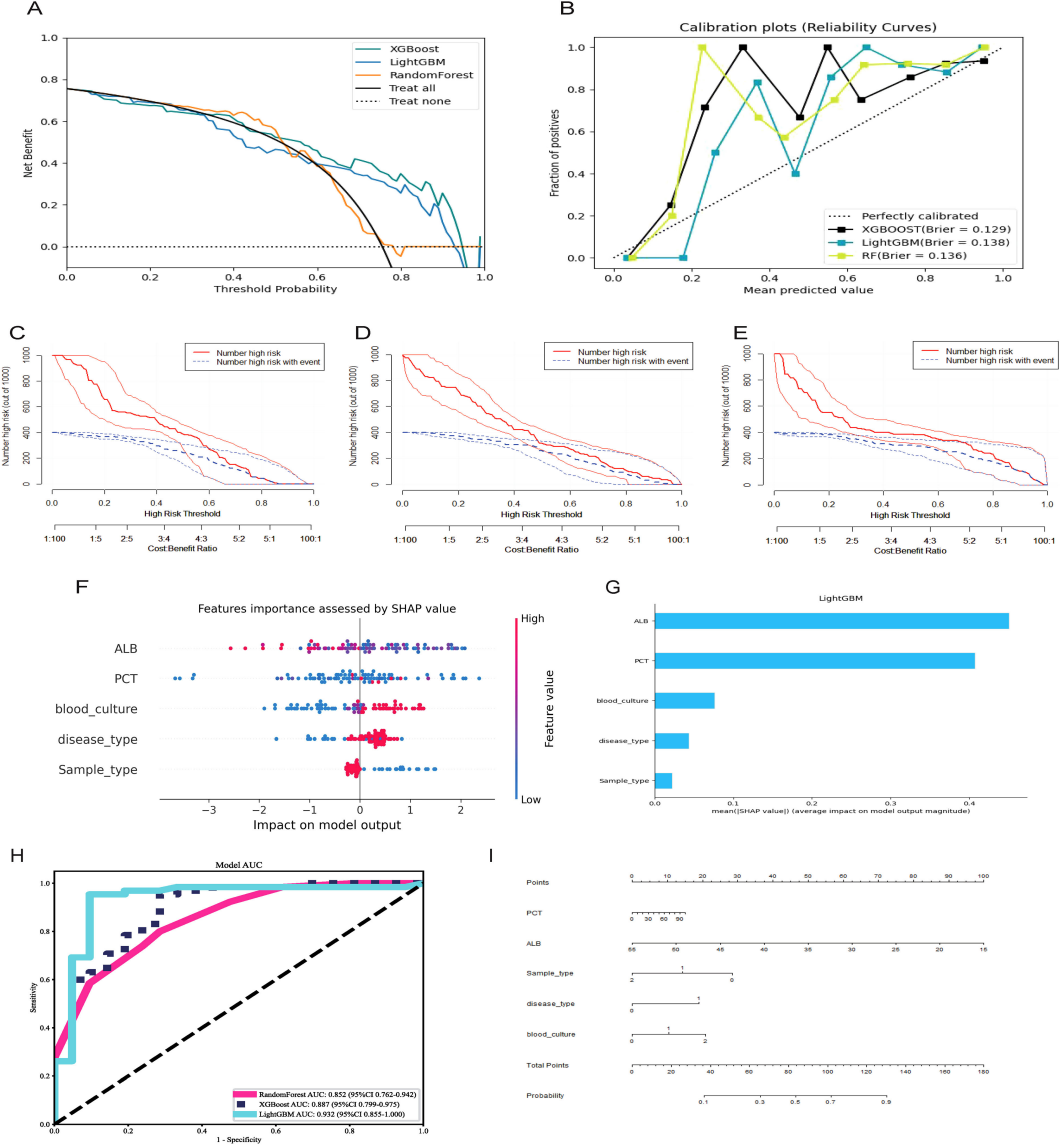
Selecting, evaluating, and building predictive models. **(A)** Decision curve analysis; **(B)** Calibration plots; **(C)** Clinical Impact Curve of XGBoost; **(D)** Clinical Impact Curve of RandomForest; **(E)** Clinical Impact Curve of LightGBM; **(F)** ROC curves for XGBoost, LightGBM, RandomForest model; **(G)** Features importance assessed by SHAP value; **(H)** Significance of the predictors in the LightGBM model; **(I)** A nomogram of the Lightgbm model for predicting mNGS results.

Furthermore, the enrolled patients were randomly assigned to the training (n = 198) and validation groups (n = 86) in a 7:3 ratio. Based on the screened key features, XGBoost, LightGBM, and RandomForest were used to predict the mNGS results. Model performance was also evaluated using DCA (**Figure 2A**: ROC Curves comparing the predictive performance of LightGBM, XGBoost, and Random Forest models), calibration curve analysis (**Figure 2B**: Calibration Curve showing the agreement between predicted probabilities and observed outcomes), and clinical impact curve analysis (**Figure 2C**: Decision Curve Analysis (DCA) demonstrating the net benefit of the predictive model across different threshold probabilities, **Figure 2D**: SHAP Summary Plot, highlighting the most important predictive features and their contribution to the model, **Figure 2E**: SHAP Dependence Plot showing the individual effect of ALB on the model’s predictions), Error bars indicate 95% confidence intervals obtained via bootstrap resampling (n=1,000 iterations) for each feature’s SHAP value. The predictive performance of the three models was relatively similar at thresholds lower than 0.6, but better performance was observed for XGBoost and LightGBM at thresholds exceeding 0.6. Concerning the calibration performance of each model, the Brier score of XGBoost was 0.129, slightly exceeding those of LightGBM (0.138) and RandomForest (0.136). As the Brier score was lower than 0.25 for all three models, the predictive probability of these models for mNGS-positive results appears to be consistent with the actual rate of occurrence. The clinical impact curve in **Figure 2** further presents the differences in model performance in predicting mNGS-positive results under different high-risk thresholds. XGBoost and RandomForest correctly identified more mNGS-positive cases at low thresholds, and their corresponding predictive accuracies were further enhanced as the threshold was increased. By contrast, LightGBM recognized fewer mNGS-positive individuals at low thresholds, but its predictive accuracy was higher than that of XGBoost and RandomForest at high thresholds.

The ROC curves and corresponding AUCs of the three ML models are presented in **Figure 2H**. The AUCs of RandomForest, XGBoost, and LightGBM were 0.852 (95% confidence interval [CI] = 0.762–0.942), 0.887 (95% CI = 0.799–0.975), and 0.932 (95% CI = 0.855–1.000), respectively. Based on an AUC close to 1, the LightGBM-based model displayed extremely high classification accuracy, and XGBoost also had excellent predictive performance. Meanwhile, RandomForest had a relatively lower AUC, but its predictive accuracy was within an acceptable range. The collective results of DCA, calibration curve analysis, clinical impact curve analysis, and ROC analysis supported the clear superiority of LightGBM.

As presented in **Table 4**, LightGBM had the best overall performance in the validation set, particularly in terms of the AUC, sensitivity, and specificity (0.93, 0.94, and 0.91, respectively). Meanwhile, RandomForest and XGBoost exhibited a certain degree of over-fitting despite excellent performance in the training set. LightGBM had better generalization performance in processing unknown data, and it was therefore considered the optimal choice among the three models. SHAP analysis was conducted to further evaluate the importance of each predictor variable in LightGBM. As presented in **Figures 2F, G**, ALB, PCT, blood culture, disease type, and sample type had the most significant impact on predicting mNGS results. In addition, a nomogram was plotted on the basis of these important features in LightGBM to predict mNGS results (**Figure 2I**). This nomogram intuitively emphasized the value of ALB as the most important predictive factor.

**Table 4:** The model evaluation of RandomForest、XGBoost and LightGBM model.

Item	Mode					
	RandomForest		XGBoost		LightGBM	
	Lable-train	Lable-test	Lable-train	Lable-test	Lable-train	Lable-test
ACC	0.97	0.74	0.90	0.88	0.73	0.93
AUC	0.99	0.85	0.97	0.89	0.84	0.93
Sensitivity	0.95	0.74	0.90	0.94	0.69	0.94
Specificity	1.00	0.76	0.91	0.71	0.79	0.91

In this study, we employed SHAP (SHapley Additive exPlanations) to interpret the predictions of our machine learning model, ensuring both explainability and robustness. Specifically, we utilized TreeExplainer, which is optimized for tree-based models such as LightGBM and XGBoost. TreeExplainer efficiently computes SHAP values by leveraging the structure of decision trees, allowing us to quantify the contribution of each feature to the model’s predictions.

To implement SHAP analysis, we calculated the marginal contribution of each feature to the prediction of mNGS positivity and examined the distribution of SHAP values to identify key factors influencing the model’s decision-making. To validate the reliability of SHAP results and ensure model robustness, we adopted the following strategies:

Comparison with Traditional Feature Importance: We compared SHAP-derived feature importance rankings with conventional importance measures (e.g., mean squared error-based rankings) to confirm the consistency of the findings.

Model Consistency Testing: We evaluated the stability of SHAP values across different random seeds and cross-validation splits, ensuring that the feature importance rankings remained stable under various data partitions.

Clinical Relevance Validation: We aligned the SHAP-identified key features (e.g., ALB, PCT) with clinical expert knowledge to ensure that the model learned meaningful and biologically relevant patterns, thereby enhancing its credibility.

SHAP analysis revealed that ALB (albumin) was the most influential predictor of mNGS test results, which aligns with the pathophysiological characteristics of infectious diseases. Additionally, PCT (procalcitonin) and blood culture results were identified as critical factors, further supporting the model’s validity. Collectively, SHAP analysis not only enhanced the interpretability of our predictive model but also provided data-driven insights to optimize mNGS testing strategies in clinical practice.

## Discussion

By detecting multiple pathogens, mNGS displays high sensitivity but low specificity in identifying the pathogens of FUO. In a prior review of nine studies, mNGS achieved positivity rates ranging from 66.7% to 93.5% for bacterial bloodstream infections and systemic infections (Marra et al., 2024). The meta-analysis results for mNGS revealed a pooled sensitivity of 0.91 (95% CI = 0.87–0.93) across three studies with a pooled specificity of 0.64 (95% CI = 0.58–0.70) (Benamu et al., 2022; Pang et al., 2023). The present study is of great significance for further optimizing the clinical application of mNGS in FUO management (Han et al., 2019).

Based on the present results, ALB, PCT, blood culture, disease type and sample type played important roles in the prediction of mNGS results. This study used the commonly used ML models XGBoost, LightGBM, and RandomForest and used mNGS positivity and negativity as binary outcomes for predictive analysis. The results confirmed the better predictive performance of the LightGBM-based model. In particular, this model exhibited stronger generalization performance when processing unknown data (Chen et al., 2024).

mNGS has high diagnostic performance, and it can be used to identify infectious pathogens that cannot be detected using traditional assays. However, it is an expensive assay, which limits its widespread clinical application. At present, there is a paucity of effective solutions for accurately predicting mNGS results, which hinders clinicians from applying mNGS in patients with FUO within a reasonable time. This lack of solutions has a direct negative impact on the diagnostic efficiency of FUO. Therefore, developing reliable predictive models to optimize the timing of mNGS in patients with FUO is of great significance for improving its diagnostic efficiency and controlling medical costs.

According to existing data, it is feasible to consider mNGS using blood samples as a first-line approach in patients with infectious FUO and assessments of specimens sampled from suspected infection sites as a second-line approach, which can significantly improve the overall diagnostic rate of FUO. This combined strategy is expected to become an optimized diagnostic solution in the future, which might improve diagnostic efficiency and accuracy and provide clinicians with more available scientific strategies for detection (Fu et al., 2021). In another study, mNGS exhibited good performance in identifying the microbiological etiology in pediatric patients with hematological malignancies accompanied by FUO. This technique can provide more extensive and accurate pathogen screening to facilitate early diagnosis and targeted treatment, providing more reliable etiological identification methods for pediatric patients with complicated conditions (Zhang et al., 2022). In recent decades, mNGS has gained popularity owing to its extraordinary pathogen diagnosis capability. However, it carries the problem of improper application timing, which transforms mNGS from a cost-effective option to an expensive alternative for detecting FUO. Consequently, there is an urgent need for an effective method to guide clinicians in determining the appropriate timing of mNGS, thereby improving its diagnostic efficiency. Based on the current findings, we expect to provide a scientific model to assist clinicians in using mNGS at the appropriate time, thereby optimizing the diagnostic process and cost-effectiveness of FUO treatment.

As indicated by our study, the common LightGBM-based ML model performed excellently in predicting mNGS results, possessing several advantages such as high efficiency, accuracy, and powerful ability to handle large-scale datasets. In multiple studies, LightGBM exhibited excellent performance, especially in medical diagnosis, making it the preferred model for processing complex clinical data precisely because of its outstanding generalization performance and short training time (Ke et al., 2017). Other researchers also constructed predictive models using six ML algorithms to predict the short-term efficacy of amlodipine in treating hypertension among inpatients. The results illustrated that the LightGBM-based ML model achieved the highest overall performance (AUC = 0.803) (Wang et al., 2024). In addition, another study predicted postoperative visual acuity in patients with epiretinal membranes undergoing vitrectomy based on a LightGBM-based model (Irie-Ota et al., 2024). Interestingly, five ML models were used to predict the etiological types of FUO. The LightGBM-based predictive model displayed the best performance, and infectious disease remained the main etiological type of classic FUO (Yan et al., 2021). Significantly, the LightGBM-based predictive model and corresponding nomogram developed in this study provide a tool to clinicians for predicting mNGS results. Based on the prediction, it is recommended to collect samples for mNGS-positive cases in a timely manner for testing, whereas for patients with negative results, other tests or a combined strategy involving multiple testing methods can be considered for comprehensive diagnosis. In this manner, the utilization of medical resources can be improved, and diagnostic efficiency can be increased to ensure the rational allocation of medical resources for diagnosis and treatment.

Several limitations of this study must be acknowledged. First, the data in this study were collected from one medical institution, which might limit the applicability of the model to a wider population. Prospective multicenter studies should be conducted to validate the findings in this study and develop predictive models with broader applicability. Second, despite the inclusion of 39 variables, our analysis did not cover all possible interfering factors of FUO. Future research should incorporate additional potential variables to further improve the model. Third, the models constructed in our study only provided binary predictions of positive and negative results without consideration of the prediction of specific pathogenic microorganisms. In the future, large-scale studies are needed to further develop a model that can predict specific pathogenic microorganisms at the mNGS level, thus improving the clinical practicality of the model. Eventually, such findings could provide more precise guidance for clinicians, assist in developing more appropriate therapeutic strategies, and optimize the clinical use of antibiotics.

We acknowledge that this study was conducted using data from a single tertiary hospital, which may limit the generalizability of the findings to other clinical settings or patient populations. While our model demonstrated strong performance in internal validation, including cross-validation and independent validation sets, external validation using datasets from other institutions has not yet been performed.

To address this limitation, future work will focus on applying the model to multicenter datasets, encompassing diverse geographical regions, healthcare systems, and patient demographics. Collaborations with external hospitals are currently under consideration to facilitate model validation on larger, heterogeneous populations. This will allow for assessment of model robustness, calibration, and potential domain shift issues when applied to different clinical environments. Furthermore, external validation could help refine feature selection and improve model adaptability, ultimately ensuring broader clinical applicability of the proposed predictive tool.

In terms of real-world deployment, the model can be embedded within existing hospital information systems (HIS) or electronic health record (EHR) platforms to provide automated clinical decision support. Upon admission, when clinicians input key patient data such as laboratory test results (e.g., albumin, procalcitonin), disease type, and sample type into the hospital system, the predictive model can automatically compute the risk score for mNGS positivity. Based on the predicted risk, the system can generate clinical alerts or recommendations, prompting physicians to either proceed with mNGS testing or consider alternative diagnostic approaches.

For practical implementation, the model can be integrated as a backend module using widely adopted programming frameworks such as Python Flask or Django, interfacing with the HIS via APIs. This will enable seamless data exchange and real-time prediction generation. To ensure clinical usability, a simple front-end dashboard or alert system can be created within the clinician workflow, displaying risk categories (e.g., high-risk vs low-risk) and visualization tools like the nomogram developed in this study.

Moreover, before full-scale deployment, the model should undergo prospective validation in multiple clinical settings to ensure reproducibility and generalizability across different patient populations. Regular model retraining using new patient data and model performance monitoring (e.g., via dashboards tracking AUC and calibration metrics) should also be established to maintain accuracy and adaptability to evolving clinical patterns.

Ultimately, the deployment of this model is expected to streamline the decision-making process, reduce diagnostic delays, and enhance the cost-effectiveness of mNGS testing in FUO management within hospital settings.

Our findings suggest that LightGBM achieved superior predictive performance compared to XGBoost and Random Forest in forecasting mNGS positivity among FUO patients. This advantage may stem from LightGBM’s unique model structure and learning mechanisms, which are particularly suited for clinical datasets characterized by high-dimensionality, sparsity, and class imbalance. Specifically, LightGBM employs gradient-based one-side sampling (GOSS) and exclusive feature bundling (EFB), enabling it to efficiently prioritize informative samples and reduce computational overhead while preserving predictive accuracy. This aligns with our dataset’s characteristics, where clinical and laboratory variables exhibit heterogeneous distributions and varying degrees of missingness.

In contrast, XGBoost—despite its effectiveness in many domains—relies on conventional histogram-based learning, which may be less optimal under conditions of sparse or imbalanced clinical data. Moreover, XGBoost showed signs of reduced generalization in the validation cohort, possibly due to its sensitivity to hyperparameter tuning within a relatively small sample size.

Random Forest, being a bagging-based ensemble method, performed comparably at lower thresholds but lagged behind LightGBM at higher probability cutoffs. This may be attributed to its inherent tendency to underfit complex interactions within clinical data when sample sizes are moderate, as observed in this study. Additionally, Random Forest produced less precise feature importance scores, making it less interpretable and actionable for clinical decision-making.

The SHAP analysis further confirmed that LightGBM not only outperformed other models in overall predictive metrics but also offered clearer insights into variable contributions. The SHAP-derived rankings identified albumin, procalcitonin, blood culture, disease type, and sample type as the most influential features—findings that are both clinically plausible and consistent with infectious disease pathophysiology.

Taken together, these observations support LightGBM as a robust and interpretable model that balances predictive power and clinical applicability, making it well suited for deployment in real-world FUO diagnostic workflows.

## Conclusion

This study constructed a predictive model based on LightGBM for the clinical prediction of mNGS results in patients with FUO and analyzed the specific feature variables (ALB, PCT, blood culture, disease type and sample type) of the model using the SHAP method. This study also plotted a nomogram based on the selected features, providing clinicians with an intuitive tool to better determine the optimal timing of mNGS. The tool developed in our study is expected to improve the efficiency of mNGS in clinical practice and optimize the diagnostic process for patients with FUO.

To translate the predictive model into clinical practice, we recommend integrating the LightGBM-based tool into the early-stage evaluation of FUO patients, particularly during infectious disease consultations or in multidisciplinary team discussions. Specifically, the model can be applied as a pre-screening tool to stratify patients based on the likelihood of mNGS positivity. For patients predicted to have a high probability of mNGS-positive results (e.g., based on nomogram scores), clinicians should prioritize mNGS testing early in the diagnostic workflow to expedite pathogen identification and reduce diagnostic delays. Conversely, for patients with low predicted probabilities, clinicians may consider alternative diagnostic strategies or adopt a stepwise approach by performing targeted microbiological tests before proceeding with mNGS.

Additionally, the model’s integration into hospital electronic health record (EHR) systems or clinical decision support systems (CDSS) could facilitate automated risk stratification, providing real-time recommendations for mNGS testing. This would not only optimize the use of mNGS resources but also reduce unnecessary testing costs, shorten hospital stays, and improve patient management efficiency.

## Data Availability

The raw data supporting the conclusions of this article will be made available by the authors, without undue reservation.
