# Supplementary material for: Enhancing fever of unknown origin diagnosis: machine learning approaches to predict metagenomic next-generation sequencing positivity
Source: Front Cell Infect Microbiol. 2025 Apr 15;15:1550933. doi: 10.3389/fcimb.2025.1550933 (PMC12037494; doi:10.3389/fcimb.2025.1550933)

**Supplemental information**

**Enhancing Fever of unknown origin Diagnosis: Machine Learning Approaches to Predict Metagenomic next-generation sequencing Positivity**

Zhi Gao**^1*,2,3^**, Yongfang Jiang**^1,2,3^**, Mengxuan Chen**^1,2,3^**, Weihang Wang**^1,2,3^**, Qiyao Liu**^1,2,3^**, Jing Ma**^1#,2,3^**


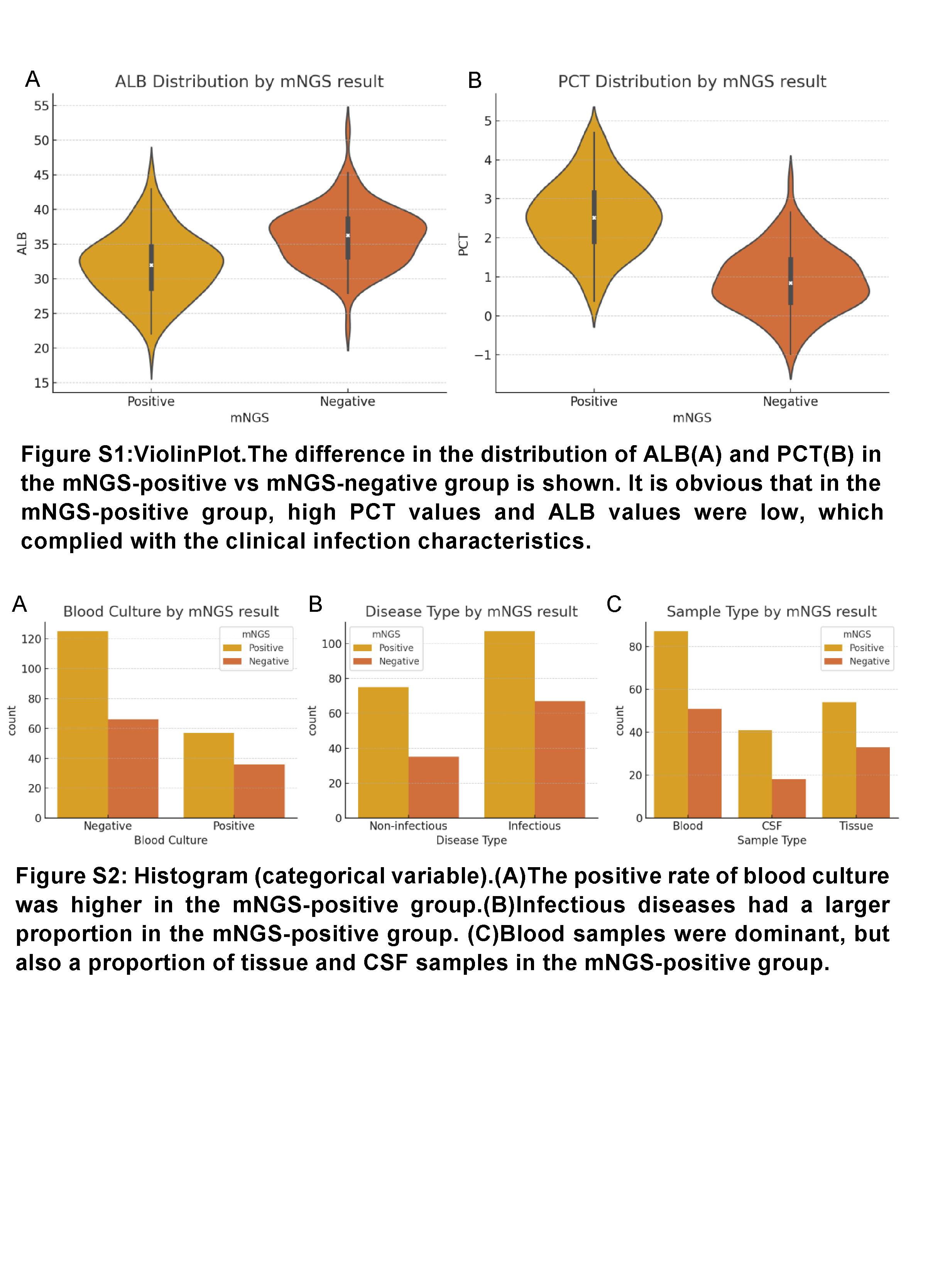

Supplement: Supplementary file 1 [file DataSheet1.docx]
